# Development of a Machine Learning-Based Triage Score for Medication-Related Osteonecrosis of the Jaw in Osteoporosis Patients Undergoing Tooth Extraction

**DOI:** 10.3390/diagnostics16121887

**Published:** 2026-06-17

**Authors:** Hui One Jeong, Cheol Won Ryu, Sung Min Park

**Affiliations:** Department of Oral and Maxillofacial Surgery, College of Dentistry, Dankook University, Cheonan 31116, Republic of Korea; jho.feb12@gmail.com (H.O.J.); dbcjfdnjs10@dankook.ac.kr (C.W.R.)

**Keywords:** medication-related osteonecrosis of the jaw, osteoporosis, antiresorptive agents, tooth extraction, machine learning, risk stratification, triage score

## Abstract

**Background/Objectives**: Medication-related osteonecrosis of the jaw (MRONJ) is a serious complication in osteoporosis patients undergoing tooth extraction. This study aimed to develop and evaluate an interpretable, machine learning–derived triage score for rapid risk stratification at the initial dental visit. **Methods**: This retrospective study included 850 osteoporosis patients (443 MRONJ, 407 controls) in the derivation cohort and 559 independent multicenter MRONJ cases for external evaluation. A reference random forest model identified a hierarchical feature structure, which was translated into an additive integer-weighted scoring system through systematic hyperparameter optimization. Structural tipping points were identified using isotonic regression and first discrete derivative analysis. Internal performance was further characterized by sensitivity, specificity, PPV, NPV, calibration slope and intercept, the Hosmer–Lemeshow test, decision curve analysis, bootstrap optimism correction, and subgroup analyses. External evaluation assessed three-tier distribution concordance and case capture rates with non-inferiority testing. **Results**: The reference random forest achieved an AUC of 0.792. The final MRONJ triage score (range 0–17) incorporated six binary predictors with mutually exclusive drug route categories. The triage score preserved discriminative performance (AUC 0.772; ΔAUC = 0.020; *p* = 0.149). Two tipping points at scores 7 and 14 defined three risk tiers: low (0–6; 20.9%), moderate (7–13; 55.3%), and high (≥14; 83.5%). At the moderate-risk threshold (≥7), the score achieved sensitivity 90.3% (95% CI 87.2–92.7%) and specificity 45.0% (40.2–49.8%); at the high-risk threshold (≥14), specificity rose to 91.4% and PPV to 83.1%. Calibration was adequate (slope 0.994; intercept 0.0006; Hosmer–Lemeshow *p* = 0.381), and decision curve analysis demonstrated higher net benefit than reference strategies across all clinically relevant threshold probabilities. The bootstrap optimism-corrected AUC was 0.778, and discriminative performance remained stable across age, route, duration, and site subgroups (AUC range 0.70–0.79). In the external cohort, the case capture rate at the ≥7 threshold was non-inferior (83.4% vs. 88.0%; Δ = −4.6%; margin −10%). **Conclusions**: The MRONJ triage score demonstrated stable discrimination and reproducible case capture in an independent multicenter cohort. By relying on six variables obtainable at the initial dental visit, this framework may have the potential to reduce unnecessary tertiary referrals and support safer clinical decision-making, although this benefit was not directly demonstrated and requires confirmation in prospective implementation studies.

## 1. Introduction

Medication-related osteonecrosis of the jaw (MRONJ) is a serious adverse event associated with antiresorptive therapy, characterized by persistent necrotic bone exposure and chronic infection in the maxillofacial region, potentially resulting in severe pain, oral functional impairment, and diminished quality of life [1]. With population aging, the expanding use of antiresorptive agents for fracture prevention in osteoporosis patients has led to a corresponding global increase in the clinical burden of MRONJ [1,2]. Osteoporosis-related MRONJ is particularly associated with dentoalveolar triggering events such as tooth extraction [1,3], and the demand for extraction remains substantial in elderly patients owing to the high global prevalence of periodontal disease and severe tooth loss [4]. Against this background, concerns regarding MRONJ risk at the time of tooth extraction in osteoporosis patients receiving antiresorptive agents have intensified, and referrals to tertiary care institutions may consequently increase.

Antiresorptive agents are the cornerstone of osteoporosis management, with fracture risk reduction well established in randomized controlled trials [5]. Although drug holidays are recommended after prolonged therapy, treatment must be resumed or switched to an alternative agent when bone mineral density is not adequately maintained or fracture risk persists during the holiday period, making discontinuation of drug exposure impractical for many patients [6,7]. Available observational data suggest that only a minority of patients temporarily discontinue medication before dentoalveolar surgery, and many remain continuously exposed to antiresorptive agents at the time of extraction or implant placement [8]. Accordingly, MRONJ risk assessment should be proactively performed within the dental care setting rather than relying solely on drug discontinuation.

Current clinical guidelines, including those of the American Association of Oral and Maxillofacial Surgeons (AAOMS), identify cumulative drug exposure, drug potency, anatomical site, and systemic comorbidities as major risk factors for MRONJ [1]. However, a simple additive clinical screening score enabling rapid risk stratification and referral decisions at the initial dental visit has not yet been established. Machine learning-based models have recently been proposed for MRONJ risk prediction [9,10]. Although such models can achieve excellent discrimination, they often entail complex input structures and limited interpretability [9,10,11,12,13,14,15,16]. Comprehensive evaluations of clinical machine learning have further emphasized that limited transparency, inconsistent reporting, and implementation complexity may constrain real-world adoption in clinical settings [11,12,13,14,17,18]. These limitations reduce practicality in general outpatient dental environments where rapid and explainable decision support is essential.

The purpose of the present study was therefore to develop and externally evaluate an interpretable additive screening score based on information obtainable at the initial dental visit.

## 2. Materials and Methods

This retrospective cohort study was conducted using electronic medical records from the Department of Oral and Maxillofacial Surgery, Dankook University Dental Hospital, covering the period from January 2019 to June 2025.

### 2.1. Patient Selection

Osteoporosis patients who had received antiresorptive agents and underwent tooth extraction at our institution, or who were referred to our institution after developing MRONJ following extraction at another facility, were included. Patients who developed MRONJ after extraction at our institution were also included. Only patients with complete records of drug type, duration, and route of administration were eligible.

An initial pool of 1031 osteoporosis patients was identified. After excluding patients with a history of head and neck radiation therapy, those with unclear drug administration duration, those receiving denosumab monotherapy without prior bisphosphonate exposure (*n* = 7; excluded due to insufficient sample size for stable stratum estimation), and those with incomplete medical or dental records, a final derivation cohort of 850 patients was established. The derivation cohort comprised 443 MRONJ patients and 407 drug-exposed controls. Within the final 850-patient analytic cohort, complete-case data were available for all 17 retained variables (0% missingness verified). This 0% missingness applied specifically to the 17 variables retained for the final analysis; clinical factors that were not retained in the final analytic dataset—most notably extraction method (simple vs. surgical) and the number of extracted teeth, which were inconsistently documented for externally referred MRONJ cases—are distinct from these retained variables and are addressed in the Limitations. Drug-exposed controls were included only if institutional records confirmed a minimum of 2 years of follow-up without MRONJ development, and MRONJ patients were included only if the onset occurred within 2 years after extraction [19]. The 2-year post-extraction window was applied based on the multicenter findings of Fung et al. [19], who reported that the median time to MRONJ onset was 5.0 months and over 90% of incident MRONJ cases manifested within 24 months of the triggering dental event. This window, therefore, captures the great majority of attributable cases while maintaining feasible follow-up windows for control ascertainment in a retrospective dataset. MRONJ was diagnosed according to the criteria set forth in the AAOMS position paper [1], and only patients with confirmed necrotic bone exposure (stage 1 or higher) were included to eliminate diagnostic ambiguity.

Each patient was represented as a single observation. Patients with multiple extraction events during the study period were represented by the index extraction—defined as the extraction triggering MRONJ onset for case patients, or the first eligible bisphosphonate-exposure extraction for control patients. Patients were uniquely identified by institutional chart number to prevent duplicate entries, and 850 unique patient identifiers were verified prior to analysis. This eliminates within-patient correlated observations and clustering effects.

### 2.2. Clinical Variables

Clinical variables were collected using a standardized questionnaire developed at our institution and administered uniformly to all eligible patients. All clinical variables (age, comorbidities, anticoagulant use, drug-administration duration, drug-administration route, and planned extraction site) were ascertained at the time of the index tooth extraction. For MRONJ cases referred from external institutions, variables were ascertained at the time of the initial extraction event preceding MRONJ development, using the referring institution’s medical records and supplementary chart review at the receiving institution. Drug-related, demographic, systemic, and local clinical variables were investigated in both the MRONJ and drug-exposed control groups. Drug-related variables included the duration and route of antiresorptive agent administration. Drug-administration duration was defined as the cumulative lifetime exposure to any antiresorptive agent—oral bisphosphonates, IV bisphosphonates, or denosumab—up to the date of the index tooth extraction. For patients with drug holidays, the cumulative exposure time included all on-treatment periods and excluded gap periods. For patients switched between agents, the total exposure across all agents was summed. Accordingly, for sequential antiresorptive exposure patients (bisphosphonate followed by switch to denosumab), drug-administration duration denotes the cumulative on-treatment time across both the bisphosphonate and the subsequent denosumab phases, rather than the duration of either agent alone. Route of administration was classified as oral bisphosphonate (PO), intravenous bisphosphonate (IV), or subcutaneous denosumab (SC). Patients who received denosumab monotherapy for osteoporosis were excluded from analysis due to insufficient case numbers (*n* = 7); consequently, all patients in the SC group had been switched from oral or intravenous bisphosphonate therapy to denosumab and were classified as the sequential antiresorptive exposure group (reflecting cumulative dual osteoclast suppression from sequential overlapping exposure rather than the intrinsic potency of a single agent). Demographic and systemic variables included age, hypertension, dyslipidemia, diabetes mellitus, cardiac disease, thyroid disease, renal disease, liver disease, rheumatoid arthritis, cancer, dementia, and anticoagulant use. Cardiovascular comorbidity in the final score was defined as the presence of hypertension and/or heart disease, reflecting the biologically plausible mechanism of vascular impairment as a unifying construct rather than a single proxy variable. Local variables included the extraction method (simple or surgical), number of extracted teeth, and extraction site (maxilla or mandible); however, because records of the extraction method and number of teeth were incomplete for externally referred patients, only the extraction site was included in the final analysis.

### 2.3. Construction of the External Case-Capture Cohort

To evaluate external validity, an independent multicenter MRONJ patient cohort was compiled from three tertiary university hospitals across South Korea (Chosun University Dental Hospital, *n* = 562; Wonkwang University Daejeon Dental Hospital, *n* = 129; Boramae Medical Center, *n* = 32). Because this cohort comprised only confirmed MRONJ cases, it was used for an external case-capture (sensitivity-focused) assessment of the score rather than a full external validation of discrimination, specificity, and calibration. The initial external dataset contained 723 confirmed MRONJ cases; after applying the same inclusion and exclusion criteria as the derivation cohort (missing drug-administration duration *n* = 164; missing extraction site *n* = 1; missing drug route *n* = 15, with substantial overlap), 559 patients were retained for the external case-capture analysis. In the external cohort, 25 of 559 MRONJ cases (4.5%) had documented involvement of both maxilla and mandible; these patients were classified as mandible-positive (mandibular site indicator = 1; 4 points) in score calculation, as the mandible is the higher-risk site. All 25 multi-site patients were retained in the external case capture analysis.

### 2.4. Machine Learning Model Development

Following data integrity verification, a complete case analysis was performed. To identify the most discriminative predictive structure, multiple supervised learning algorithms were trained on the derivation cohort, including logistic regression (linear baseline), random forest, gradient boosting machine (GBM), and extreme gradient boosting (XGBoost) [20,21,22]. All candidate algorithms were implemented in Python (version 3.13; scikit-learn 1.8.0; XGBoost 3.1.3) with a fixed random seed (random state = 42) for reproducibility, and the external dataset was withheld from all model training and tuning. Model performance was estimated by stratified 5-fold cross-validation (shuffled, seed = 42) using the area under the receiver operating characteristic curve (AUC) as the evaluation metric [23]. The reference random forest, from which the variable-importance hierarchy was derived, used 500 trees (n_estimators = 500) with balanced class weighting to account for outcome distribution; the remaining settings followed scikit-learn defaults (no maximum depth, min_samples_split = 2, min_samples_leaf = 1, and max_features set to the square root of the predictor count). For the gradient boosting machine and XGBoost, package-default settings were used (gradient boosting: 100 estimators, learning rate 0.1, maximum depth 3; XGBoost: 100 estimators, learning rate 0.3, maximum depth 6, evaluated with the logistic-loss objective). Logistic regression used L2 regularization with balanced class weighting.

Among the evaluated models, the random forest classifier demonstrated the highest and most stable cross-validated discriminative performance and was therefore selected as the reference predictive model. This model served to identify the most robust predictive signals and to establish a performance benchmark for the subsequent construction of the additive screening score. Model development and reporting followed established methodologies and recent precedents in machine learning-based clinical prediction [12,14,24].

### 2.5. Construction of the Additive Triage Scoring System

Variable importance was quantified using the mean decrease in Gini impurity within the random forest framework; predictors contributing the greatest cumulative reduction in classification impurity were ranked as the most informative features and selected for score construction [20].

Drug administration duration and age were initially modeled as continuous predictors. To preserve discrimination while enabling an interpretable score-based system, candidate dichotomization thresholds for each variable were evaluated using systematic hyperparameter optimization and cross-validated AUC. For drug administration duration, thresholds from 1 to 5 years were tested, and the ≥3-year cutpoint was selected as the final threshold because it preserved discrimination relative to the continuous model and minimized performance variance. For age, thresholds of 65, 70, 75, and 80 years were examined, and the ≥70-year cutpoint was adopted as the final threshold because it maintained cross-validated discrimination with limited information loss.

Integer weights were assigned to each binary predictor through systematic hyperparameter optimization with the dual objective of approximating the reference random forest model’s performance while maintaining clinical interpretability [25]. For each of the six binary predictors, a predefined candidate integer-weight range was specified based on the rank position of mean decrease in Gini impurity from the reference random forest model: drug-administration duration ≥ 3 years [candidate range: 4–7]; mandibular extraction site [3–5]; sequential antiresorptive exposure [2–4]; cardiovascular comorbidity (hypertension and/or heart disease) [1–3]; age ≥ 70 years [1–3]; standard intravenous bisphosphonate administration [0–2]. All feasible integer combinations within these ranges (4 × 3 × 3 × 3 × 3 × 3 = 972 candidate configurations) were systematically evaluated against three concurrent criteria: (i) stratified 5-fold cross-validated AUC ≥ reference random forest − 0.03; (ii) inter-fold AUC standard deviation ≤ 0.05; and (iii) strictly monotonic risk gradient across observed score strata. For each candidate configuration, discrimination (AUC), inter-fold performance stability, and risk monotonicity across score strata were simultaneously assessed. Among the 36 configurations satisfying all three criteria, the final weights (6, 4, 3, 2, 2, 1) were selected because they minimized the absolute AUC deviation from the reference model while preserving integer parsimony and clinical interpretability, and were assigned as follows; drug-administration duration ≥ 3 years, 6 points; mandibular extraction site, 4 points; sequential antiresorptive exposure, 3 points; cardiovascular comorbidity (hypertension and/or heart disease), 2 points; age ≥ 70 years, 2 points; and standard intravenous bisphosphonate administration, 1 point. Although these nominal weights sum to 18, the two drug-route predictors—sequential antiresorptive exposure (3 points) and standard intravenous bisphosphonate administration (1 point)—are mutually exclusive, because each patient is assigned to only a single administration route (oral, intravenous, or subcutaneous) and these two predictors therefore cannot be simultaneously assigned to the same patient. Consequently, the maximum attainable total score is 17 points (obtained when sequential antiresorptive exposure is present), and a score of 16 is structurally unreachable. Notably, the candidate-range upper bounds (4–7, 3–5, 2–4, 1–3, 1–3, 0–2; nominal sum 24) describe only the search space evaluated during optimization and do not correspond to attainable score totals.

### 2.6. Identification of Structural Tipping Points and Risk Stratification

A two-stage approach was applied to identify structural tipping points in the observed MRONJ proportion across total score levels. In the primary analysis, score strata with no observed patients or structurally unreachable scores were excluded from analysis, and strata with fewer than 10 observations (0 < *n* < 10) were excluded from isotonic regression fitting in accordance with the National Center for Health Statistics (NCHS) data presentation standards for proportions and replaced by linear interpolation between adjacent retained strata [26]. Sample-size–weighted isotonic regression (pool-adjacent-violators algorithm) was then fitted to the remaining strata under a monotonicity constraint to yield a monotone-smoothed risk curve across the score range [27,28].

Tipping points were identified by analyzing the first discrete derivative of the monotone-smoothed curve—that is, the single-step increment (Δ%) in fitted risk between adjacent scores—and transitions exhibiting the largest Δ% were designated as candidate structural tipping points defining risk tier boundaries.

As a supplementary analysis to confirm the robustness of the tipping points, adjacent scores were merged into fixed-width 3-point bins [29], and Wilson 95% confidence intervals were computed. Bin width was determined after comparing 2-point, 3-point, and 4-point widths with simultaneous consideration of monotonicity preservation, minimum bin sample size, and resolution for detecting gradient changes. Based on the identified tipping points, the MRONJ triage score was stratified into clinical risk tiers, and tier-specific MRONJ event rates were calculated in the derivation cohort.

To assess the robustness of the chosen cutoffs, a sensitivity analysis was performed across nine alternative (low, high) cutoff pairs combining low-risk thresholds of 6, 7, or 8 with high-risk thresholds of 13, 14, or 15. The resulting sensitivity, specificity, PPV and tier distribution metrics were compared with those of the original (7,14) cutoffs to evaluate robustness of the chosen thresholds.

### 2.7. Statistical Analysis

Continuous variables were reported as medians with interquartile ranges (IQR) and compared using the Mann–Whitney U test. Categorical variables were presented as frequencies and percentages and compared using the chi-square test or Fisher’s exact test [30]. Internal validation employed stratified 5-fold cross-validation in the derivation cohort. Because each observation was predicted exactly once across the five folds, the pooled out-of-fold predictions constituted a single non-redundant set suitable for paired comparison; discriminative comparison between the reference random forest model and the MRONJ triage score was accordingly performed using the DeLong test on these pooled predictions [23]. To complement cross-validated estimates and address potential optimism from repeated weight optimization, Harrell’s bootstrap optimism correction was performed with 200 bootstrap replications, and a nested 5 × 5 cross-validation was implemented for the reference random forest (outer folds for testing, inner folds for hyperparameter selection). Multicollinearity was assessed by computing variance inflation factors (VIF) and Spearman correlation matrices for the six binary score predictors. Calibration was evaluated using calibration slope, calibration-in-the-large (intercept), the Hosmer–Lemeshow goodness-of-fit test, and decile-grouped calibration plots, after logistic recalibration of the integer score into predicted probabilities. Decision curve analysis was performed over threshold probabilities of 0.05–0.95 with comparison against treat-all and treat-none reference strategies. At pre-specified score thresholds (≥7 and ≥14 points), the full confusion matrix and sensitivity, specificity, positive predictive value (PPV), negative predictive value (NPV), and likelihood ratios were computed with Wilson 95% confidence intervals. Subgroup discrimination was evaluated by computing the score AUC within strata defined by age (<70 vs. ≥70 years), drug-administration route, drug-administration duration (<3 vs. ≥3 years), and extraction site, with 95% confidence intervals derived from 200 bootstrap replications. Because the external cohort comprised only MRONJ-positive cases, external specificity and AUC could not be estimated. External validation was assessed by concordance of three-tier risk distribution between the derivation cohort MRONJ patients and the external cohort (χ^2^ test, Cramér’s V) [30] and by case capture rates at pre-specified thresholds (≥7 and ≥14 points) with Wilson 95% confidence intervals. For the case capture rate at the moderate-risk threshold (≥7 points), a pre-specified non-inferiority margin of −10% was applied, with non-inferiority declared if the lower bound of the 95% confidence interval for the between-cohort difference exceeded −10% [31]. All analyses were performed using Python (version 3.13; scikit-learn version 1.8.0 and XGBoost version 3.1.3) and R (version 4.5.2; pROC version 1.19.0.1 and isotone version 1.1-2). The study is reported in accordance with the Transparent Reporting of a multivariable prediction model for Individual Prognosis or Diagnosis (TRIPOD) guidelines. A two-sided *p* < 0.05 was considered statistically significant.

## 3. Results

### 3.1. Study Population

**Figure 1:**
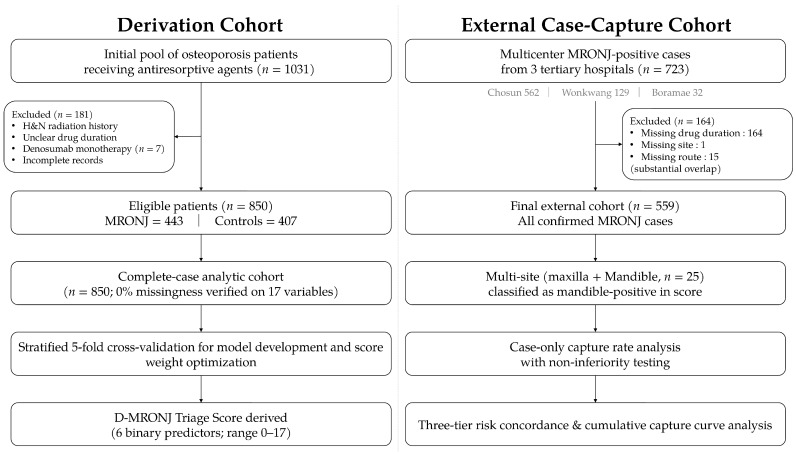
Patient selection flowchart for the derivation and external cohorts. The left panel shows the stepwise exclusion process and analytic workflow for the derivation cohort (final *n* = 850; 443 MRONJ cases, 407 controls). The right panel shows case selection for the external case-capture cohort (final *n* = 559 confirmed MRONJ cases) and the subsequent case-capture analyses.

A total of 1409 patients were included, comprising 850 in the derivation cohort (443 MRONJ patients and 407 drug-exposed controls) and 559 in the external MRONJ cohort. The full patient selection flow is shown in Figure 1. In the derivation cohort, MRONJ patients were older than controls, had longer antiresorptive agent exposure, and had a higher prevalence of mandibular extraction and hypertension. The median age was 78 years (IQR 72–83) in the MRONJ group versus 75 years (IQR 66–81) in the control group (*p* < 0.001), and the proportion aged ≥70 years was 83.3% versus 64.6%, respectively (*p* < 0.001). Long-term antiresorptive therapy (≥3 years) was present in 77.0% of the MRONJ group compared with 39.6% of controls (*p* < 0.001). The external MRONJ cohort exhibited broadly similar baseline characteristics to the derivation cohort MRONJ cases with respect to age, drug administration duration, and mandibular extraction (Table 1).

### 3.2. Reference Random Forest Model Performance

A random forest classifier was trained on the derivation cohort (*n* = 850) using stratified 5-fold cross-validation [20]. The AUC computed by pooling out-of-fold predictions across the five folds was 0.792 (95% CI: 0.762–0.822), demonstrating robust discriminative performance. This result served as the performance benchmark for the subsequent development of the additive MRONJ triage score.

Comparative cross-validated performance of all candidate algorithms is presented in Table 2. Logistic regression achieved 0.778 ± 0.046; random forest, 0.791 ± 0.035; gradient boosting, 0.786 ± 0.029; and XGBoost, 0.773 ± 0.024. Random forest yielded the highest mean AUC and was therefore selected as the reference model. Robustness analyses showed that nested 5 × 5 cross-validation for the reference random forest produced an AUC of 0.791 ± 0.035, essentially identical to the standard 5-fold estimate, confirming that the reported internal-validation estimates are not artificially inflated. Bootstrap optimism correction for the final integer score is reported separately in Section 3.4.

### 3.3. Variable Ranking and Score Configuration

Variable importance analysis based on mean decrease in Gini impurity from the reference random forest model revealed a consistent hierarchical structure, with variables ranked in descending order of importance as follows: drug administration duration, mandibular extraction site, sequential antiresorptive exposure, cardiovascular comorbidity (hypertension and/or heart disease), age, and injection route (standard intravenous bisphosphonate). This ranking was stable across all five cross-validation folds.

In the dichotomization threshold evaluation, the ≥3-year cutpoint for drug administration duration was selected because it simultaneously preserved discrimination and minimized inter-fold performance variance among candidates ranging from ≥1 to ≥5 years. For age, the ≥70-year cutpoint demonstrated optimal cross-validated discrimination among candidates of 65, 70, 75, and 80 years and was therefore adopted.

Exhaustive evaluation of all feasible integer weight combinations within predefined ranges [25] identified a final configuration that simultaneously satisfied discrimination (AUC), inter-fold stability, and risk monotonicity across score strata. The final MRONJ triage score assigned 6 points for drug administration duration ≥ 3 years, 4 points for mandibular extraction site, 3 points for sequential antiresorptive exposure (combined/switched bisphosphonate-to-denosumab therapy), 2 points for cardiovascular comorbidity (hypertension and/or heart disease), 2 points for age ≥ 70 years, and 1 point for standard intravenous bisphosphonate administration, yielding a maximum attainable total score of 17 points (Table 3).

In support of the 1-point weight for standard IV bisphosphonate (in contrast to the 3-point weight for sequential antiresorptive exposure), within-derivation MRONJ event rates stratified by route showed a clear gradient. Within patients with cumulative drug exposure ≥ 3 years (the clinically relevant high-risk stratum), observed rates were: oral bisphosphonate 63.5%, standard IV bisphosphonate 71.4%, and sequential antiresorptive exposure 80.0%. The intermediate rate for IV bisphosphonate justified the intermediate integer weight (Table 4).

### 3.4. Internal Performance of the MRONJ Triage Score

**Figure 2:**
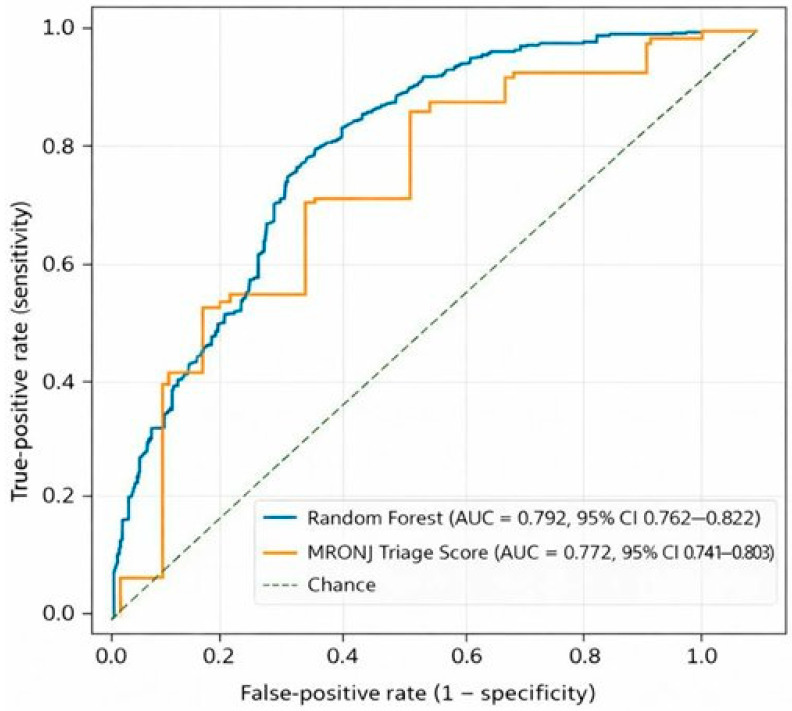
Receiver operating characteristic (ROC) curves of the reference random forest model and the MRONJ triage score in the derivation cohort (*n* = 850). The reference random forest achieved an AUC of 0.792 (95% CI 0.762–0.822), and the MRONJ triage score achieved an AUC of 0.772 (95% CI 0.741–0.803). The difference was not statistically significant (ΔAUC = 0.020; DeLong *p* = 0.149).

The AUC of the MRONJ triage score, computed by pooling stratified 5-fold cross-validated out-of-fold predictions, was 0.772 (95% CI: 0.741–0.803), indicating that the discriminative performance of the reference random forest model was substantially preserved (ΔAUC = 0.020; *p* = 0.149, DeLong test) (Figure 2).

Bootstrap optimism correction (200 replications) yielded an optimism of +0.001 for the integer score, with an optimism-corrected AUC of 0.778—essentially identical to the apparent estimate—confirming that the integer-based scoring approach is intrinsically resistant to overfitting.

Confusion matrices and associated diagnostic metrics at the two pre-specified score thresholds are summarized in Table 5. At the ≥7 (moderate-risk) threshold, the score achieved sensitivity 90.3% (95% CI 87.2–92.7%), specificity 45.0% (40.2–49.8%), PPV 64.1% (60.3–67.8%), and NPV 81.0% (75.4–85.6%); positive likelihood ratio 1.64; negative likelihood ratio 0.22. At the ≥14 (high-risk) threshold, sensitivity was 38.8% (34.4–43.4%), specificity 91.4% (88.3–93.8%), PPV 83.1% (77.4–87.6%), and NPV 57.9% (54.0–61.6%); positive likelihood ratio 4.51; negative likelihood ratio 0.67. These figures confirm a two-tier clinical decision architecture: the ≥7 threshold prioritizes sensitivity for case capture and referral triggering, while the ≥14 threshold prioritizes specificity and PPV for rule-in/urgent specialist consultation.

### 3.5. Calibration and Decision Curve Analysis

**Figure 3:**
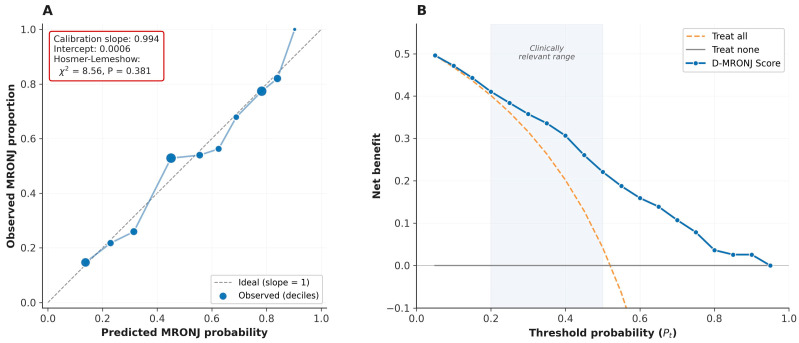
Calibration and decision curve analysis of the MRONJ triage score in the derivation cohort (*n* = 850). (**A**) Calibration plot. Predicted MRONJ probabilities, obtained by logistic recalibration of the integer score, were grouped into deciles (blue circles; marker size proportional to within-decile sample size) and plotted against the observed MRONJ proportion. The dashed grey diagonal indicates perfect calibration; the inset reports calibration slope, intercept, and Hosmer–Lemeshow goodness-of-fit statistics. (**B**) Decision curve analysis. Net benefit is shown across threshold probabilities (Pt) for the D-MRONJ triage score (solid blue), ‘treat all’ (dashed orange), and ‘treat none’ (grey). The light-blue shaded region (Pt = 0.20–0.50) denotes the clinically relevant threshold range for tooth-extraction referral decisions. Pt, threshold probability.

Calibration of the D-MRONJ score, evaluated after logistic recalibration into predicted probabilities, demonstrated near-ideal agreement between predicted and observed MRONJ rates. The calibration slope was 0.994 (ideal = 1.0) and the calibration-in-the-large intercept was 0.0006 (ideal = 0). The Hosmer–Lemeshow goodness-of-fit test was not statistically significant (χ^2^ = 8.56, df = 8, *p* = 0.381), supporting adequate calibration (Figure 3A). Observed-versus-expected MRONJ rates by risk tier showed close agreement: low-risk tier 19.0% observed vs. 19.2% expected; moderate-risk tier 54.7% vs. 54.8%; high-risk tier 83.1% vs. 82.7%. Decision curve analysis (Figure 3B) demonstrated that the D-MRONJ score yielded higher net benefit than both ‘treat-all’ and ‘treat-none’ reference strategies across all clinically plausible threshold probabilities (Pt = 0.05–0.95). At a representative referral threshold of Pt = 0.30, net benefit for the score was 0.358 vs. 0.316 (treat-all) vs. 0 (treat-none); at Pt = 0.50, net benefit was 0.221 vs. 0.042 vs. 0. These findings support the score’s net clinical utility in real-world referral decision-making.

### 3.6. Observed Risk Gradient and Structural Tipping Points

**Figure 4:**
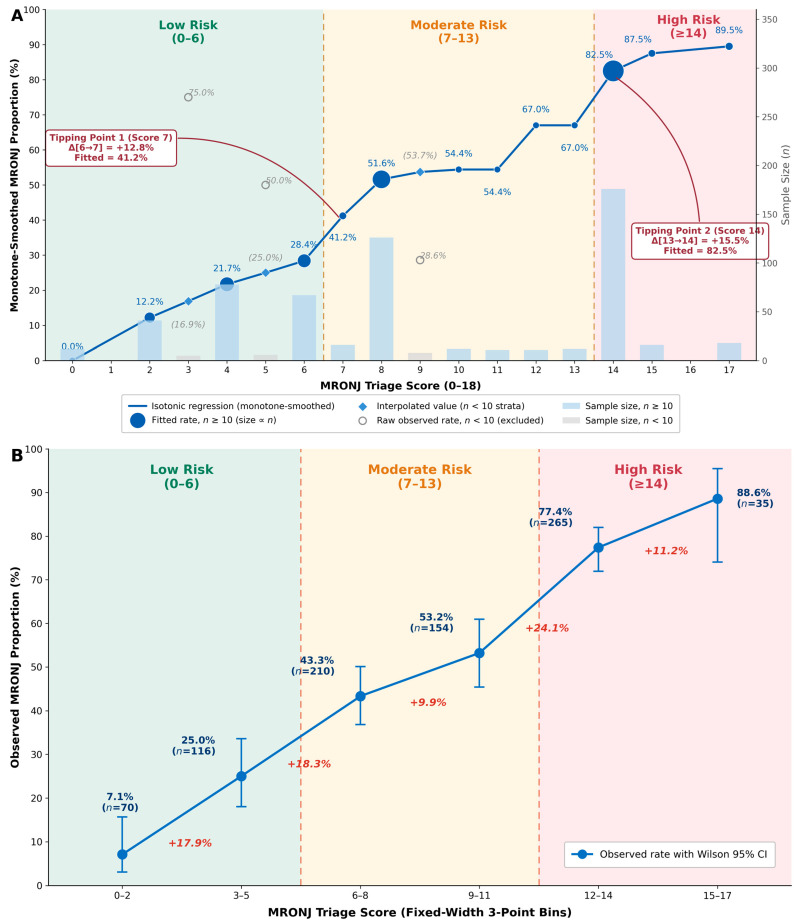
Observed risk gradient and structural tipping points of the MRONJ triage score (derivation cohort, *n* = 850). (**A**) Isotonic regression of MRONJ proportions across score levels. Score 1 (*n* = 0) was excluded, and score 16 is omitted from the abscissa because it is structurally unreachable: the 3-point weight for sequential antiresorptive exposure and the 1-point weight for standard intravenous bisphosphonate are mutually exclusive (drug routes are categorical), so no combination of weights can sum to exactly 16. Three strata with *n* < 10 (score 3, *n* = 4; score 5, *n* = 6; score 9, *n* = 7; gray open circles) were replaced by linear interpolation (italic parenthetical values). Tipping points were identified by the maximum first discrete derivative of the monotone-smoothed curve: score 7 (Δ[6 → 7] = +12.8%) and score 14 (Δ[13 → 14] = +15.5%), defining low-risk (0–6), moderate-risk (7–13), and high-risk (≥14) tiers. Blue bars indicate sample size per score level (secondary axis). (**B**) Fixed-width 3-point bin analysis with Wilson 95% confidence intervals. Inter-bin increments corroborate the tipping point locations (3–5 → 6–8: Δ = +18.3%; 9–11→12–14: Δ = +24.1%).

The score distribution in the derivation cohort spanned from 0 to 17 points, with one score stratum having no observed patients (score 1; *n* = 0) and score 16 being structurally unreachable due to the mutual exclusivity of drug route weights. Three strata had sample sizes below 10 (score 3, *n* = 4, observed proportion 75.0%; score 5, *n* = 6, observed proportion 50.0%; score 9, *n* = 7, observed proportion 28.6%). Strata with no observed patients or structurally unreachable scores (scores 1 and 16) were excluded from analysis, and strata with *n* < 10 were excluded from isotonic regression fitting in accordance with NCHS data presentation standards and replaced by linear interpolation between adjacent large-sample strata (score 3: 75.0% → 16.9%; score 5: 50.0% → 25.0%; score 9: 28.6% → 53.7%) [26]. Interpolated estimates fell naturally between the observed proportions of adjacent large-sample strata. After applying sample-size–weighted isotonic regression to the remaining 13 retained strata, MRONJ proportions increased monotonically from 0.0% at score 0 to 89.5% at score 17 (Figure 4A).

Analysis of the first discrete derivative (Δ%) of the monotone-smoothed curve identified two distinct gradient accelerations. In the lower score range, the largest increment was observed at the transition from score 6 to score 7 (Δ[6 → 7] = +12.8%; fitted proportion 28.4% → 41.2%), designating score 7 as the first tipping point separating the low-risk trajectory (0–6 points) from the moderate-risk tier (7–13 points). In the upper score range, the largest single increment across the entire score range was observed between scores 13 and 14 (Δ[13 → 14] = +15.5%; fitted proportion 67.0% → 82.5%), designating score 14 as the second tipping point marking the entry boundary of the high-risk tier (≥14 points). Although these two transitions were close in magnitude to the third-ranked transition (Δ[11 → 12] = +12.6%), the same locations were independently confirmed in the subsequent 3-point bin supplementary analysis, supporting the tipping point selection (Figure 4A).

The fixed-width 3-point bin supplementary analysis confirmed the same hierarchical structure [29]: 0–2 points, 7.1% (95% CI: 3.1–15.7%); 3–5 points, 25.0% (18.0–33.6%); 6–8 points, 43.3% (36.8–50.1%); 9–11 points, 53.2% (45.4–61.0%); 12–14 points, 77.4% (71.9–82.0%); 15–17 points, 88.6% (74.0–95.5%). The inter-bin increments flanking the tipping points (3–5 → 6–8: Δ = +18.3%; 9–11 → 12–14: Δ = +24.1%) corresponded to the locations of the first and second tipping points, respectively (Figure 4B).

### 3.7. Three-Tier Risk Stratification of the MRONJ Triage Score

**Figure 5:**
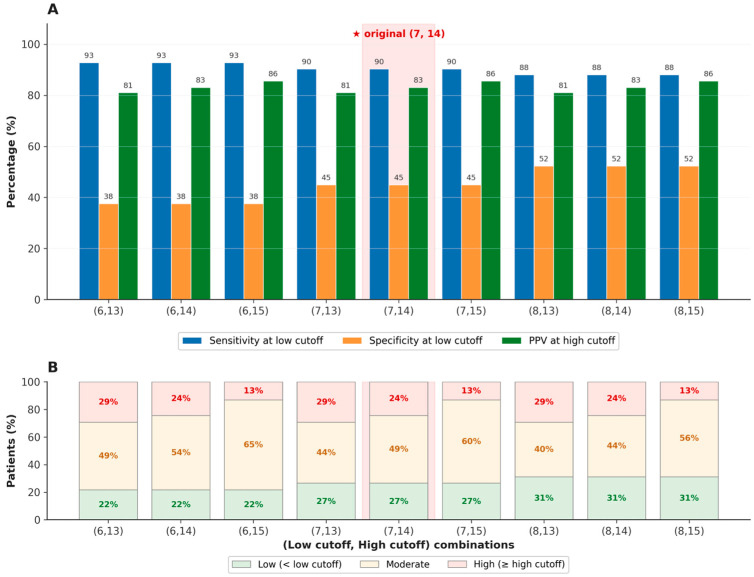
Alternative cutoff sensitivity analysis of the MRONJ triage score. Nine candidate (low, high) cutoff combinations were evaluated to assess the robustness of the chosen tier boundaries; the original (7, 14) used in the main analysis is highlighted with red background shading and a red star. (**A**) Sensitivity at the low cutoff (blue), specificity at the low cutoff (orange), and positive predictive value (PPV) at the high cutoff (green) across all nine combinations. (**B**) Tier distribution under each combination: low-risk (green; below the low cutoff), moderate-risk (yellow; between cutoffs), and high-risk (pink; at or above the high cutoff). Within-tier MRONJ event-rate monotonicity (low < moderate < high) was preserved across all nine combinations, supporting the robustness of the three-tier risk structure. PPV, positive predictive value.

Based on these data-driven tipping points, the MRONJ triage score was stratified into three clinical risk tiers: low risk (0–6 points), moderate risk (7–13 points), and high risk (≥14 points). In the derivation cohort, MRONJ event rates demonstrated stepwise accumulation across tiers: low risk 20.9% (53/253), moderate risk 55.3% (213/385), and high risk 83.5% (177/212) (Cochran-Armitage test for trend, *p* < 0.001) (Table 6). A sensitivity analysis across nine alternative (low, high) cutoff combinations confirmed that tier monotonicity (low < moderate < high) was preserved across all combinations, with the original (7, 14) cutoffs providing the optimal balance of sensitivity at the screening threshold and PPV at the rule-in threshold (Table 7 and Figure 5).

### 3.8. Subgroup Performance

Subgroup analyses (Table 8) confirmed stable discriminative performance of the D-MRONJ score across major clinically relevant strata. Subgroup AUC ranged from 0.698 to 0.789: age < 70 years 0.774 (95% CI 0.696–0.829); age ≥ 70 years 0.756 (0.722–0.794); oral bisphosphonate 0.763 (0.720–0.808); IV bisphosphonate 0.786 (0.740–0.832); sequential antiresorptive exposure 0.789 (0.689–0.866); drug duration < 3 years 0.713 (0.654–0.765); drug duration ≥ 3 years 0.698 (0.644–0.745); mandibular site 0.759 (0.712–0.801); maxillary site 0.739 (0.687–0.797). The slightly lower AUC within the duration ≥ 3-year subgroup reflects ceiling-effect dynamics—nearly all such patients fall into the moderate or high-risk tier—rather than degraded discrimination. Sex was not included as a candidate predictor and sex-stratified subgroup AUCs were not computed, for three convergent reasons. First, sex showed no significant association with MRONJ in the derivation cohort (male 5.4% in controls vs. 3.4% in MRONJ cases; χ^2^ *p* = 0.149; Table 1), and it ranked lowest among the evaluated variables in the random forest mean-decrease-in-Gini importance hierarchy, well below the six predictors retained for the score. Second, the cohort was markedly female-predominant (93.6–96.6%), leaving only 36 male patients across both cohorts—too few for stable patient-level subgroup AUC estimation. Third, sex was recorded only as an aggregate cohort-level descriptor (Table 1) and was not retained as a case-level analyzable field in the de-identified analytic dataset. The exclusion of sex, therefore, reflects both its lack of predictive contribution and the structural limitations of the cohort, rather than an oversight.

### 3.9. External Case-Only Evaluation

**Figure 6:**
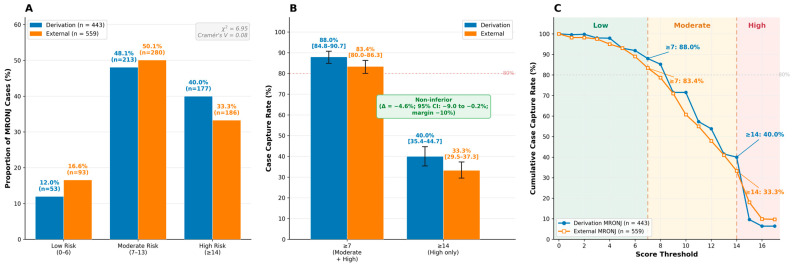
External case-only validation of the MRONJ triage score. (**A**) Risk tier distribution of confirmed MRONJ cases in the derivation (*n* = 443) and external (*n* = 559) cohorts. The three-tier structure was concordant across cohorts (χ^2^ = 6.95; Cramér’s V = 0.08, small effect size). (**B**) Case capture rates at the moderate-risk (≥7) and high-risk (≥14) thresholds with Wilson 95% confidence intervals. At the ≥7 threshold, a pre-specified non-inferiority margin of −10% was satisfied (Δ = −4.6%; 95% CI of difference: −9.0% to −0.2%). The dashed line indicates the 80% reference threshold. (**C**) Cumulative case capture curves across all score thresholds, demonstrating close alignment between the two independent cohorts. Vertical dashed lines denote the tipping point boundaries (scores 7 and 14).

External evaluation was performed on 559 independent multicenter MRONJ cases. The three-tier risk distribution was broadly concordant with that of the derivation cohort MRONJ patients (χ^2^ = 6.95; Cramér’s V = 0.08, small effect size) (Figure 6A). The case capture rate at the moderate-risk threshold (≥7 points) was maintained from 88.0% (95% CI: 84.8–90.7%) in the derivation cohort to 83.4% (95% CI: 80.0–86.3%) in the external cohort, with the difference falling within the pre-specified non-inferiority margin of −10% (Δ = −4.6%; 95% CI of difference: −9.0% to −0.2%) (Figure 6B). The cumulative case capture curves demonstrated close alignment of trajectories between the two cohorts across the full score range, confirming that the discriminative structure of the score was reproduced in the independent cohort (Figure 6C).

## 4. Discussion

In the present study, an explainable additive screening score for MRONJ was developed in osteoporosis patients receiving antiresorptive agents. Using a structured machine learning-based framework, the hierarchical feature structure identified by a random forest model was translated into an interpretable integer-based scoring system suitable for decision-making at the initial dental visit.

Machine learning approaches are particularly valuable in biomedical contexts characterized by nonlinear interactions and complex multivariate dependencies [32,33]. However, the contemporary clinical AI literature emphasizes that discrimination alone is insufficient for implementation in high-stakes medical decision-making, and that interpretability, transparency, and workflow integration are essential for clinical adoption [34,35,36]. Accordingly, rather than deploying a black-box classifier, model-derived feature importance was translated into a transparent additive score consistent with modern principles of clinically transferable predictive modeling [37,38].

### 4.1. Mutual Exclusivity and Attainable Range of the Score Structure

Of the six binary predictors weighted at 6, 4, 3, 2, 2, and 1 points respectively (Table 3), sequential antiresorptive exposure (3 points) and standard IV bisphosphonate (1 point) are derived from the mutually exclusive drug route variable, while the remaining predictors are independent. Because drug administration routes were classified as mutually exclusive categories—oral (PO), intravenous (IV), and subcutaneous (SC; combined/switched)—the 3 points for sequential antiresorptive exposure and the 1 point for intravenous bisphosphonate administration could not be simultaneously assigned to the same patient. Consequently, the maximum attainable total score was 17 points, and a score of 16 represented a structural gap that was unreachable under any combination of route-specific weights.

Importantly, the MRONJ triage score was conceived as a screening tool rather than a probabilistic prediction model. Traditional prediction models aim to estimate absolute event probabilities and therefore require rigorous calibration assessment and external transportability evaluation [37,38,39]. In contrast, screening tools are designed to minimize missed cases of disease [40]. Because MRONJ can result in a prolonged treatment course and a potentially unfavorable prognosis once established, the consequences of failing to detect an at-risk patient are far more serious than the clinical cost of an unnecessary referral. It is therefore clinically reasonable to prioritize case capture performance over absolute event probability estimation [40,41].

The score demonstrated internally stable discrimination (AUC 0.772; 95% CI: 0.741–0.803), with no statistically significant difference from the reference random forest model (ΔAUC = 0.020; *p* = 0.149). External evaluation was conducted as a case-only capture assessment on 559 independent multicenter MRONJ cases. The inclusion of only positive cases in the external cohort reflected the fact that MRONJ can develop with a delayed onset ranging from several weeks up to 2 years after extraction [19], making it practically difficult to assemble controls with confirmed ≥2-year follow-up and complete drug exposure records across multiple institutions. In particular, inconsistent documentation of drug administration route and cumulative duration across institutions precluded the assembly of a control group comparable to that of the derivation cohort. Under these constraints, a sensitivity-focused evaluation strategy is methodologically justified when the primary objective of the screening tool is case detection rather than probability estimation [40,41,42].

Because the external cohort included only confirmed MRONJ cases, external specificity, AUC, and calibration could not be estimated. The external evaluation should therefore be interpreted as a case-only capture (sensitivity-focused) validation rather than as a comprehensive external validation, and absolute external risk estimates derived from this study should be interpreted with caution. Prospective full case–control external validation incorporating both MRONJ cases and drug-exposed controls is required before clinical adoption.

In external validation, the three-tier risk distribution was broadly concordant with that of the derivation cohort MRONJ patients (χ^2^ = 6.95; Cramér’s V = 0.08, small effect size), and the case capture rate at the moderate-risk threshold (≥7 points) was maintained within the pre-specified non-inferiority margin of −10% (Δ = −4.6%; 95% CI of difference: −9.0% to −0.2%). The cumulative case capture curves also demonstrated close alignment of trajectories between the two cohorts across the full score range (Figure 6). In general, prediction models tend to experience performance degradation when applied to external populations with different clinical characteristics, attributable to differences in disease prevalence, patient composition, and care setting [38,43,44]. The finding that the discriminative structure of the score was preserved despite the external cohort having been collected from independent multicenter settings suggests that the screening function was not overfit to the characteristics of a single institution [40].

### 4.2. Methodological Considerations in Tipping Point Identification

The validity of tipping point-based risk stratification is supported by three methodological lines of evidence.

First, with respect to the handling of sparse strata: one score stratum with no observed patients (score 1) and the structurally unreachable score of 16 were excluded from analysis, and three strata with 0 < *n* < 10 (score 3, *n* = 4; score 5, *n* = 6; score 9, *n* = 7) were excluded from isotonic regression fitting based on NCHS data presentation standards and replaced by linear interpolation between adjacent retained strata [26]. Linear interpolation is a standard approach for handling sparse observation points in isotonic regression [27,28], and the interpolated estimates fell naturally between the observed proportions of adjacent large-sample strata, maintaining monotonicity.

Second, the tipping point locations converged with the natural cluster boundaries of patient density. Because four of six variables carry even-numbered weights (6, 4, 2, and 2 points), 88.1% of all patients were concentrated at even-numbered scores, with prominent density peaks at scores 4 (*n* = 106), 8 (*n* = 126), 10 (*n* = 127), 12 (*n* = 76), and 14 (*n* = 177). The first tipping point (score 7) was located at the natural boundary between the low-risk concentration zone (scores 4–6) and the moderate-risk concentration zone (scores 8–10–12), and the second tipping point (score 14) coincided with the highest-density score and the point at which the MRONJ event rate surged to 82.5%. This convergence of structural transitions in the risk gradient and cluster boundaries in patient density suggests that the tipping points reflect clinically distinct risk profile transitions rather than statistical artifacts.

Third, the robustness of the tipping points was independently confirmed in the fixed-width 3-point bin analysis [29]. Three-point bins (6 bins, minimum *n* = 35) offered an optimal balance between Wilson confidence interval stability and gradient resolution compared with 2-point bins (residual per-bin instability) and 4-point bins (insufficient resolution). The inter-bin increments flanking the tipping points (3–5 → 6–8: Δ = +18.3%; 9–11 → 12–14: Δ = +24.1%) independently confirmed the locations identified by the discrete derivative analysis.

A further sensitivity analysis (Table 7) tested nine alternative (low, high) cutoff combinations spanning (6, 13) to (8, 15). Tier monotonicity was preserved across all nine combinations, and the original cutoffs (7, 14) provided a balanced trade-off between sensitivity at the screening threshold (90.3%) and PPV at the rule-in threshold (83.1%). Although the discrete-derivative magnitudes at the 6 → 7 (+12.8%) and 11 → 12 (+12.6%) transitions are close, the convergent evidence from patient-density cluster boundaries and the independent 3-point bin analysis specifically supports score 7—not score 12—as the structural transition.

### 4.3. Consistency with the Existing Literature

The hierarchical structure of the score is broadly consistent with established pathophysiological understanding and epidemiological evidence for MRONJ.

Drug administration duration. The highest weight (6 points) for drug administration duration (≥3 years) is consistent with evidence of a strong time-dependent relationship with MRONJ risk. MRONJ risk in oral bisphosphonate users is negligible during the first 3 years but increases significantly beyond 4 years [1,45,46]. This reflects the pharmacokinetic properties of nitrogen-containing bisphosphonates, which bind with high affinity to bone hydroxyapatite and accumulate in the jawbone over the years, resulting in cumulative osteoclast suppression, impaired microcrack repair, and diminished regenerative capacity [1,47,48]. Multiple systematic reviews have confirmed long-term antiresorptive therapy as the most important risk factor for MRONJ in osteoporosis patients [49,50].

Mandibular extraction site. The high weight (4 points) for the mandibular site is consistent with reports that MRONJ occurs approximately 2 to 3 times more frequently in the mandible than in the maxilla [1,47,51,52]. This predilection is attributed to the mandible’s limited vascular supply via a terminal vasculature configuration, higher cortical bone ratio with preferential bisphosphonate accumulation, and greater biomechanical loading in the posterior region [1,47,53,54].

Sequential antiresorptive exposure. The 3 points for sequential exposure to multiple antiresorptive agents reflect evidence that combined or switched therapy significantly increases MRONJ risk. Srivastava et al. [55] reported a weighted MRONJ prevalence of 13% in the sequential bisphosphonate-to-denosumab group, versus 5% for bisphosphonate monotherapy and 4% for denosumab monotherapy. This is explained by overlapping dual suppression of osteoclast function [1,55,56,57]. Recent meta-analyses have further demonstrated that denosumab carries a higher MRONJ risk than zoledronic acid at equivalent follow-up intervals [58], and prospective data confirm substantially elevated incidence in high-dose denosumab recipients [59]. Within-derivation route-stratified event rates (Table 4) showed that among patients with ≥3 years of cumulative exposure, MRONJ rates were 63.5% for oral bisphosphonate, 71.4% for standard IV bisphosphonate, and 80.0% for sequential antiresorptive exposure, supporting the relative weights of 0, 1, and 3 points, respectively.

Age. Age (≥70 years, 2 points) is an established risk factor, with systematic reviews reporting mean ages of 69 to 72 years in MRONJ patients [1,9]. The increased risk in elderly patients reflects immunosenescence, reduced stem cell regenerative capacity, microvascular degeneration, and longer cumulative antiresorptive exposure [1,9,49].

Cardiovascular comorbidity. Hypertension and heart disease, jointly weighted (2 points) as cardiovascular comorbidity, have been identified as systemic risk factors for MRONJ in multiple studies [9,60]. Proposed mechanisms include altered calcium metabolism, chronic endothelial dysfunction, and microvascular compromise that may compound the vascular impairment induced by antiresorptive agents in the jawbone [61,62]. Multicollinearity assessment confirmed a variance inflation factor of 1.13 for cardiovascular comorbidity with a Spearman correlation of 0.33 with age ≥ 70 years, indicating no problematic collinearity. More importantly, the 2-point weight equivalent to age ≥ 70 years was justified by two structural considerations. First, the variable importance ranking from the reference random forest, which reflects each predictor’s marginal contribution within the multivariable context rather than its univariate effect size, ranked cardiovascular comorbidity as the 4th most informative predictor—above age ≥ 70 years (5th). Second, the systematic grid-search optimization across 972 candidate weight configurations (Section 2.5) selected 2 points as the value that minimized inter-fold AUC variance while preserving the monotonicity criterion. Together, these analyses support the inclusion and equivalent weighting of cardiovascular comorbidity as an independent score component.

Intravenous administration route. The minimum weight (1 point) for standard intravenous bisphosphonate (zoledronic acid 5 mg/year) reflects its intermediate risk profile—higher than oral formulations due to complete systemic bioavailability, but substantially lower than oncologic high-dose regimens (zoledronic acid 4 mg/month) [1,45,47]. The intermediate within-derivation MRONJ rate of 71.4% (in patients with ≥3 years cumulative exposure), positioned between PO (63.5%) and sequential exposure (80.0%), supports this 1-point integer weight.

In summary, the six-item hierarchical structure demonstrates that machine learning–derived variable importance is consistently aligned with established MRONJ pathophysiology, epidemiological risk factor structure, and clinical guideline recommendations. This convergence strengthens the construct validity of the score and supports the balance between data-driven prediction and clinical interpretability.

### 4.4. Effect of Case Enrichment and Referral Patterns on Performance Estimates

Several features of the study design warrant explicit interpretation. By construction, the derivation cohort was a matched case-control sample of confirmed MRONJ cases and drug-exposed controls rather than a consecutive clinical series, producing an MRONJ prevalence of 52.1% that greatly exceeds the true incidence among osteoporosis patients undergoing tooth extraction. This enrichment has direct and predictable consequences for several reported metrics. Because positive and negative predictive values depend on prevalence, the PPVs observed here, and the absolute MRONJ rates assigned to each risk tier (20.9%, 55.3%, and 83.5%), are specific to the enriched sampling frame and will be lower in a routine, low-prevalence population; they should therefore be read as relative risk-stratification levels rather than as transportable absolute probabilities. Discrimination metrics (AUC, sensitivity) are comparatively more stable across prevalence but remain subject to spectrum bias, because the case–control design samples cases and controls from the extremes of the clinical spectrum and may overstate separability relative to an unselected population.

The same caution applies to the calibration and decision-curve results. The near-ideal calibration slope (0.994) and intercept (0.0006) were obtained by logistic recalibration of the integer score within the enriched derivation cohort itself; they demonstrate internal coherence of the score-to-probability mapping but do not establish that predicted probabilities will be well calibrated in an external low-prevalence setting, where systematic over-prediction would be expected without re-estimation of the intercept. Likewise, the favorable net benefit in the decision-curve analysis is conditioned on the enriched outcome frequency and should be interpreted as internal supportive evidence rather than a population-level estimate of clinical utility. A further structural consideration is that confirmed MRONJ cases were frequently referred from external institutions, whereas drug-exposed controls were drawn from local follow-up records. This asymmetry introduces the potential for referral and information bias: referred cases and locally managed controls may differ in documentation completeness and in the distribution of predictors, which could accentuate apparent differences between groups. We mitigated this by ascertaining predictors at the time of the index extraction using referring-institution records and supplementary chart review, and by restricting the final score to variables that were uniformly documented across sites; nevertheless, residual differences in data capture cannot be excluded and may have influenced predictor distributions and estimated performance. These considerations reinforce that the present results establish internal validity and a reproducible discriminative structure, while transportable absolute risks, external calibration, and real-world utility require prospective validation in consecutive primary-care populations.

### 4.5. Clinical Implications

Beyond biological consistency, this screening framework addresses system-level challenges. In the absence of structured risk stratification, precautionary referral to tertiary institutions for drug-exposed patients requiring extraction is common. Although prudent, this strategy increases patient travel burden, wait times, and specialist workload, and may contribute to inefficient allocation of healthcare resources [63,64]. Digital clinical decision support systems have been demonstrated to improve referral appropriateness and optimize resource utilization in other areas of healthcare [65,66]. By stratifying risk at the initial dental visit using routinely obtainable variables, the MRONJ triage score may have the potential to reduce unnecessary tertiary referrals while maintaining high sensitivity for clinically significant risk. We emphasize, however, that this is a hypothesized benefit inferred from the score’s discriminative behavior in a retrospective dataset; it was not directly demonstrated by the present study design. Whether the score actually reduces referrals or improves decision-making can only be established by prospective impact studies that evaluate clinical implementation and downstream outcomes.

Clinical example. A 76-year-old woman with a 5-year history of oral alendronate therapy and well-controlled hypertension presents to her general dentist for planned extraction of a non-restorable mandibular molar (#46). Score calculation: duration ≥ 3 years (+6) + mandibular site (+4) + cardiovascular comorbidity (+2) + age ≥ 70 years (+2) + sequential antiresorptive exposure (0) + standard IV bisphosphonate (0; route is oral) = total 14 points. This patient falls into the high-risk tier (≥14), corresponding to an internally observed MRONJ rate of 83.5%. The clinical recommendation is direct referral to a tertiary specialist for surgical planning and pre-extraction risk mitigation. By contrast, a 65-year-old woman with 2 years of oral bisphosphonate therapy planning a maxillary extraction with no cardiovascular comorbidity would score 0 (low risk; 20.9% observed rate), supporting management in primary dental care.

Decision curve analysis (Figure 3B) confirms that the score provides higher net benefit than ‘treat-all’ or ‘treat-none’ reference strategies across all clinically plausible threshold probabilities, indicating a favorable theoretical net benefit; because this analysis is itself derived from the enriched retrospective cohort, it should be regarded as supportive rather than confirmatory evidence of real-world referral utility, pending prospective evaluation [67].

## 5. Limitations

The present study has several limitations.

First, the retrospective design introduces the possibility of unmeasured confounding variables. Clinical variables were collected using a standardized institutional questionnaire, and exclusions were primarily attributable to incomplete drug exposure records rather than random missingness; however, the complete-case analysis employed may introduce selection bias if data were not missing completely at random. Although the derivation cohort included 443 events across 6 binary predictors—yielding approximately 74 events per variable, which exceeds recommended minimums for stable binary prediction models [44]—the relatively small absolute sample and the integer-based scoring approach make validation in larger and more diverse populations desirable.

Second, both cohorts were assembled at tertiary referral institutions and the external cohort comprised only confirmed MRONJ cases, which precluded estimation of external specificity, AUC, and calibration and rendered the derivation prevalence case-enriched (52.1%). Consequently, the tier-specific absolute rates (20.9%, 55.3%, 83.5%) and discriminative metrics are affected by case-control sampling and spectrum bias and should be read as relative-risk stratification rather than population incidence, with rank-ordering being the more transferable property of the score. Nevertheless, the concordance in risk-tier distribution (Cramér’s V = 0.08), non-inferiority at the moderate-risk threshold, and close alignment of cumulative capture curves indicate that the score was not overfit to the development institution. Because the intended use is screening and referral rather than probability estimation, prioritizing case capture is consistent with its purpose; prospective full case-control validation in primary dental care is nonetheless required to recalibrate absolute risks and confirm the transportability of the ≥7 and ≥14 thresholds [37,38,40,41,42,43].

Third, in the tipping point identification, the first-ranked (Δ[13→14] = +15.5%), second-ranked (Δ[6→7] = +12.8%), and third-ranked (Δ[11→12] = +12.6%) transitions were close in magnitude. Nevertheless, the convergence of three independent lines of evidence—patient-density cluster boundaries, isotonic regression with sparse-stratum handling, and the fixed-width 3-point bin analysis (Section 4.2)—specifically supported score 7 (not score 12) as the lower tipping point and score 14 as the upper tipping point. The (7, 14) cutoffs should nevertheless be reassessed in independent cohorts to confirm threshold robustness.

Fourth, the cohort was 94.6–96.6% female, reflecting the underlying epidemiology of osteoporosis. Generalizability to male patients with osteoporosis or with malignancy-driven bone metastasis—populations with distinct comorbidity profiles and antiresorptive prescribing patterns—remains to be established [6].

Fifth, the 24-month post-extraction window for MRONJ outcome ascertainment may have misclassified a small number of delayed-onset cases (>24 months) as drug-exposed controls. Because such misclassification reduces, rather than inflates, apparent performance, the reported metrics may underestimate the score’s true discriminative ability [19].

Sixth, several clinically plausible factors were not incorporated into the final score. Low-frequency systemic comorbidities (liver disease 1.2%, kidney disease 3.5%, rheumatoid arthritis 5.6%, dementia 1.2%, chemotherapy history 0.7%) may have failed to reach significance owing to type II error rather than a true absence of association, and local surgical factors—extraction method (simple vs. surgical) and the number of extracted teeth—were not retained because they were inconsistently documented for externally referred cases. In addition, a small number of patients (*n* = 7) receiving denosumab monotherapy without prior bisphosphonate exposure were excluded for insufficient sample size, so the score applies to patients with a bisphosphonate exposure history (with or without subsequent switch to denosumab) and should not be extrapolated to bisphosphonate-naïve denosumab recipients. Future studies using larger, prospectively collected cohorts with complete local-factor documentation should reassess these factors and validate the score in denosumab-monotherapy populations [10,13,44,53,58].

## 6. Conclusions

The MRONJ triage score is an explainable, additive screening and referral tool for osteoporosis patients receiving antiresorptive agents. Developed through machine learning-based feature selection and interpretability optimization, the score demonstrated stable internal discrimination (AUC 0.772), adequate calibration (slope 0.994; intercept 0.0006; Hosmer–Lemeshow *p* = 0.381), and favorable decision-curve net benefit across all clinically plausible thresholds. Robustness was confirmed through bootstrap optimism correction (corrected AUC 0.778), nested cross-validation, and consistent discriminative performance across age, route, duration, and site subgroups. External case capture performance was maintained (83.4% at the ≥7-point threshold), with reproducibility in an independent multicenter external cohort confirmed through non-inferiority testing and cumulative capture curve analysis.

By relying on only six variables obtainable at the initial dental visit, this screening framework may have the potential to reduce unnecessary tertiary referrals, improve specialist resource allocation, and support safer clinical decision-making for tooth extraction in osteoporosis patients; however, this potential benefit was not directly demonstrated by the present retrospective design and requires prospective confirmation. Prospective full case-control external validation incorporating both MRONJ cases and drug-exposed controls in primary dental care settings is required to confirm threshold transportability, external calibration, and system-level impact before broad clinical adoption.

## Figures and Tables

**Table 1:** Baseline characteristics of the derivation and external cohorts.

Variables	Control (Non-MRONJ) (*n* = 407)	MRONJ (Case) (*n* = 443)	*p*-Value	External MRONJ-Only (*n* = 559)
Sex, *n* (%)			0.149 ^2^	
Male	22 (5.4)	15 (3.4)	–	36 (6.4)
Female	385 (94.6)	428 (96.6)	–	523 (93.6)
Age, years				
Median (IQR)	75 (66–81)	78 (72–83)	<0.001 ^1^	77 (72–82)
≥70 years, *n* (%)	263 (64.6)	369 (83.3)	<0.001 ^2^	454 (81.2)
Medication duration				
Median (IQR), years	2 (0–4)	5 (3–9)	<0.001 ^1^	5 (3–8)
≥3 years, *n* (%)	161 (39.6)	341 (77.0)	<0.001 ^2^	357 (63.9)
Medication type, *n* (%)				
Oral bisphosphonates	199 (48.9)	213 (48.1)	0.866 ^2^	239 (42.8)
IV bisphosphonates	176 (43.2)	173 (39.1)	0.242 ^2^	197 (35.2)
Sequential antiresorptive exposure ^†^	32 (7.9)	57 (12.9)	0.023 ^2^	123 (22.0)
Extraction site, *n* (%)				
Maxilla	205 (50.4)	113 (25.5)	<0.001 ^2^	145 (25.9)
Mandible	202 (49.6)	330 (74.5)	<0.001 ^2^	414 (74.1) ^‡^
Comorbidities, *n* (%)				
Hypertension	195 (47.9)	295 (66.6)	<0.001 ^2^	353 (63.1)
Heart disease	41 (10.1)	79 (17.8)	0.001 ^2^	91 (16.3)
Hyperlipidemia	75 (18.4)	111 (25.1)	0.013 ^2^	109 (19.5)
Diabetes mellitus	76 (18.7)	112 (25.3)	0.025 ^2^	140 (25.0)
Anticoagulant use	70 (17.2)	96 (21.7)	0.088 ^2^	69 (12.3)
Thyroid disease	31 (7.6)	32 (7.2)	0.819 ^2^	33 (5.9)
Liver disease	5 (1.2)	5 (1.1)	0.928 ^2^	8 (1.4)
Kidney disease	10 (2.5)	20 (4.5)	0.094 ^2^	17 (3.0)
Rheumatoid arthritis	17 (4.2)	31 (7.0)	0.102 ^2^	31 (5.5)
Cancer history	24 (5.9)	35 (7.9)	0.250 ^2^	83 (14.8)
Dementia	4 (1.0)	6 (1.4)	0.632 ^2^	13 (2.3)
Chemotherapy history	1 (0.2)	5 (1.1)	0.101 ^2^	49 (8.8)

Abbreviations: MRONJ, medication-related osteonecrosis of the jaw; IQR, interquartile range; IV, intravenous. ^†^ Sequential antiresorptive exposure denotes patients who received bisphosphonate therapy followed by a switch to denosumab, reflecting cumulative dual osteoclast suppression rather than the intrinsic potency of a single agent. Patients receiving denosumab monotherapy without prior bisphosphonate exposure were excluded from the derivation cohort due to insufficient sample size (*n* = 7). *p*-values were calculated between the Control and MRONJ groups within the derivation cohort. ^1^ Mann–Whitney U test; ^2^ Chi-square test. Values are presented as median (IQR) or *n* (%). ^‡^ In the external cohort, 25 of 559 MRONJ cases had documented involvement of both maxilla and mandible; these patients were classified as mandible-positive in score calculation and were retained in the case capture analysis.

**Table 2:** Candidate model performance comparison (5-fold cross-validation).

Model	Mean AUC	SD	Range	Notes
Logistic regression (L2)	0.778	0.046	0.743–0.841	Baseline linear model
Random forest (*n* = 500)	0.791	0.035	0.767–0.852	Reference model (final)
Gradient boosting	0.786	0.029	0.750–0.823	Sklearn default hyperparams
XGBoost	0.773	0.024	0.746–0.802	Default hyperparameters
D-MRONJ score (integer)	0.779	-	0.749–0.811 *	Deterministic; * = bootstrap range

All algorithms trained on the same 17-variable derivation cohort (*n* = 850). Except for the reference random forest (n_estimators = 500 with balanced class weighting), default package hyperparameters were used; full settings are reported in Section 2.4. Values in this table are fold-mean AUC ± standard deviation across the five cross-validation folds; the reference random forest AUC of 0.792 cited in the main text was instead computed by pooling out-of-fold predictions across folds, which is the more appropriate single-estimate metric and differs marginally from the fold-mean (0.791) by construction.

**Table 3:** MRONJ triage score: components and point assignment.

Variable	Criterion	Points
Medication duration	≥3 years	6
Extraction site	Mandible	4
Sequential antiresorptive exposure ^†^	Combined bisphosphonate-to-denosumab therapy	3
Cardiovascular comorbidity	Hypertension and/or heart disease	2
Age	≥70 years	2
Standard IV bisphosphonates	Intravenous administration route	1
Total score range		0–17

MRONJ, medication-related osteonecrosis of the jaw; IV, intravenous. ^†^ Point weights were derived from a grid search over 972 candidate integer weight combinations, 36 of which satisfied predefined criteria for discrimination (AUC), fold-to-fold stability, and risk monotonicity across score strata.

**Table 4:** Route-specific MRONJ event rates, stratified by drug-administration duration.

Drug Route	Duration < 3 Years	Duration ≥ 3 Years	Overall
Oral bisphosphonate	37/135 (27.4%)	176/277 (63.5%)	213/412 (51.7%)
Standard IV bisphosphonate	48/174 (27.6%)	125/175 (71.4%)	173/349 (49.6%)
Sequential antiresorptive exposure	17/39 (43.6%)	40/50 (80.0%)	57/89 (64.0%)

Within the high-risk stratum (duration ≥ 3 years), the MRONJ rate for standard IV bisphosphonate (71.4%) is intermediate between oral bisphosphonate (63.5%) and sequential antiresorptive exposure (80.0%), supporting the 1-point integer weight assigned to IV BP. Note that absolute rates reflect case-enriched ascertainment in a tertiary referral institution and should be interpreted as relative-risk-stratification probabilities, not as population incidence.

**Table 5:** Performance metrics at key score thresholds (derivation cohort, *n* = 850).

Metric	Threshold ≥ 7 (Moderate Risk)	Threshold ≥ 14 (High Risk)
True positives (TP)	400	172
False positives (FP)	224	35
False negatives (FN)	43	271
True negatives (TN)	183	372
Sensitivity	90.3% (87.2–92.7)	38.8% (34.4–43.4)
Specificity	45.0% (40.2–49.8)	91.4% (88.3–93.8)
PPV	64.1% (60.3–67.8)	83.1% (77.4–87.6)
NPV	81.0% (75.4–85.6)	57.9% (54.0–61.6)
Positive LR	1.64	4.51
Negative LR	0.22	0.67

Values in parentheses are 95% Wilson confidence intervals. PPV, positive predictive value; NPV, negative predictive value; LR, likelihood ratio.

**Table 6:** Three-tier risk stratification of the MRONJ triage score in the derivation cohort.

Risk Category	Score Range	Total Patients *N* (%)	MRONJ Events N	MRONJ Rate, % (95% CI)
Low risk	0–6	253 (29.8)	53	20.9 (16.3–26.4)
Moderate risk	7–13	385 (45.3)	213	55.3 (50.3–60.2)
High risk	≥14	212 (24.9)	177	83.5 (77.8–88.0)
Total	0–17	850 (100)	443	52.1 (48.7–55.5)

Three-tier risk stratification of the MRONJ triage score in the derivation cohort (*n* = 850). Risk categories were defined by structural tipping points at scores 7 and 14. 95% CI, Wilson score method.

**Table 7:** Alternative cutoff sensitivity analysis.

(Low, High)	*N* (Low)	*N* (Mod)	*N* (High)	Sens@Low	Spec@Low	PPV@High	Tier Monotonicity
(6, 13)	185	417	248	92.8%	37.6%	81.0%	Preserved
(6, 14)	185	458	207	92.8%	37.6%	83.1%	Preserved
(6, 15)	185	554	111	92.8%	37.6%	85.6%	Preserved
(7, 13)	226	376	248	90.3%	45.0%	81.0%	Preserved
(7, 14) *	226	417	207	90.3%	45.0%	83.1%	Preserved (original)
(7, 15)	226	513	111	90.3%	45.0%	85.6%	Preserved
(8, 13)	266	336	248	88.0%	52.3%	81.0%	Preserved
(8, 14)	266	377	207	88.0%	52.3%	83.1%	Preserved
(8, 15)	266	473	111	88.0%	52.3%	85.6%	Preserved

* = original cutoffs used in the manuscript. All nine candidate (low, high) combinations preserve strict tier monotonicity (low < moderate < high MRONJ rate). The original (7, 14) combination provides a balanced trade-off: high sensitivity (90.3%) at the screening threshold and high PPV (83.1%) at the rule-in threshold.

**Table 8:** Subgroup analyses (derivation cohort, *n* = 850).

Subgroup	*n*	MRONJ %	AUC	95% CI	Sens@≥7/Spec@≥7
Age < 70 years	218	33.9%	0.774	0.696–0.829	71.6%/72.2%
Age ≥ 70 years	632	58.4%	0.756	0.722–0.794	94.0%/30.0%
Oral bisphosphonate	412	51.7%	0.763	0.720–0.808	89.7%/48.2%
IV bisphosphonate	349	49.6%	0.786	0.740–0.832	89.0%/46.6%
Sequential antiresorptive exposure	89	64.0%	0.789	0.689–0.866	96.5%/15.6%
Drug duration < 3 years	348	29.3%	0.713	0.654–0.765	61.8%/69.9%
Drug duration ≥ 3 years	502	67.9%	0.698	0.644–0.745	98.8%/6.8%
Mandibular extraction site	532	62.0%	0.759	0.712–0.801	94.8%/28.7%
Maxillary extraction site	318	35.5%	0.739	0.687–0.797	77.0%/61.0%

AUC values were computed using the integer D-MRONJ score on each subgroup. 95% confidence intervals are bootstrap percentile intervals (200 replications). Sex-stratified subgroup AUCs were not computed because sex was non-significant (χ^2^ *p* = 0.149), ranked lowest in random forest variable importance, and was available only as an aggregate cohort-level descriptor (Table 1) rather than a case-level analyzable field; the small male count (*n* = 36 across both cohorts) further precluded stable estimation.

## Data Availability

The data that support the findings of this study are available from the corresponding author upon reasonable request due to privacy and ethical restrictions, as they contain sensitive personal health information of patients collected under institutional review board approval that does not permit public deposition. For the multicenter external cohort, data sharing is additionally subject to the data-sharing agreements of the participating institutions. Access is subject to institutional review board approval.

## Abbreviations

The following abbreviations are used in this manuscript:

MRONJ	Medication-related osteonecrosis of the jaw
AAOMS	American Association of Oral and Maxillofacial Surgeons
GBM	Gradient boosting machine
XGBoost	Extreme gradient boosting
AUC	Area under the receiver operating characteristic curve
NCHS	National Center for Health Statistics
IQR	Interquartile range
TRIPOD	Transparent Reporting of a Multivariable Prediction Model for Individual Prognosis or Diagnosis
VIF	Variance inflation factor
PPV	Positive predictive value
NPV	Negative predictive value
LR	Likelihood ratio
DCA	Decision curve analysis
CV	Cross-validation
